# 贝叶斯方法在肿瘤新药早期临床研发中的发展与应用

**DOI:** 10.3779/j.issn.1009-3419.2022.102.43

**Published:** 2022-10-20

**Authors:** 慧瑶 黄, 梅若 刘, 喜艳 李, 鑫雨 孟, 丹丹 崔, 烨 冷, 玉 唐, 宁 李

**Affiliations:** 1 100021 北京，国家癌症中心/国家肿瘤临床医学研究中心/中国医学科学院北京协和医学院肿瘤医院，药物临床试验研究中心 Clinical Trials Center, National Cancer Center/National Clinical Research Center for Cancer/Cancer Hospital, Chinese Academy of Medical Sciences and Peking Union Medical College, Beijing 100021, China; 2 065001 廊坊，国家癌症中心/国家肿瘤临床医学研究中心/河北中国医学科学院肿瘤医院，药物临床试验研究中心 Clinical Trials Center, National Cancer Center/National Clinical Research Center for Cancer/Hebei Cancer Hospital, Chinese Academy of Medical Sciences, Langfang 065001, China; 3 201203 上海，勃林格殷格翰(中国)投资有限公司数据分析与统计部门 Department of Biostatistics and Data Sciences, Boehringer Ingelheim (China) Investment Co Ltd, Shanghai 201203, China; 4 3010 维多利亚州，墨尔本大学公共卫生学院 School of Population and Global Health, the University of Melbourne, Victoria 3010, Australia; 5 211198 南京，中国药科大学基础医学与临床药学系 School of Basic Medicine and Clinical Pharmacy, China Pharmaceutical University, Nanjing 211198, China

**Keywords:** 早期试验, 贝叶斯, 统计设计, 肿瘤, Exploratory trial, Bayesian, Statistical design, Neoplasm

## Abstract

贝叶斯学派是通过综合未知参数的先验信息与样本信息，依据贝叶斯定理，求出后验分布，根据后验分布推断未知参数的统计方法。相比频率派，贝叶斯学派更加灵活、高效。肿瘤新药是全球研发的热点，但同时也存在高失败率的风险。在肿瘤新药早期研发中，高效寻找最佳剂量、优势人群、估计疗效和成功率是医药企业和研究者的共同需求。近年来，肿瘤新药研发呈现化学药物生物制品转变、单药治疗向联合治疗转变、传统设计向创新设计转变等新趋势; 伴随出现的各种挑战，包括无法找到最高耐受剂量、延迟毒性、延迟反应、剂量疗效关系变化、剂量组合众多等。基于贝叶斯方法，恰当借用先验信息，能有效帮助企业在肿瘤早期研发中，实现从传统研发模式(高投入、长周期、低效率)向现代研发模式(低投入、短周期、高效率)的转变。研究还进行了贝叶斯方法在肿瘤新药早期研发的进展阐述，与频率派的理念、应用场景的比较分析，可为医药研发的所有从业人员提供宏观、系统的参考。

## Abstract (en)

Bayesian statistics is an approach for learning from evidences as it accumulates, combining prior distribution with current information on a quantity of interest, in which posterior distribution and inferences are being updated each time new data become available using Bayes' Theorem. Though frequentist approach has dominated medical studies, Bayesian approach has been more and more widely recognized by its flexibility and efficiency. Research and development (R & D) on anti-cancer new drugs have been so hot globally in recent years in spite of relatively high failure rate. It is the common demand of pharmaceutical enterprises and researchers to identify the optimal dose, regime and right population in the early-phase R & D stage more accurately and efficiently, especially when the following three major changes have been observed. The R & D on anticancer drugs have transformed from chemical drugs to biological products, from monotherapy to combination therapy, and the study design has also gradually changed from traditional way to innovative and adaptive mode. This also raises a number of subsequent challenges on decision-making of early R & D, such as inability to determine MTD, flexibility to deal with delayed toxicity, delayed response and dose-response changing relationships. It is because of the above emerging changes and challenges that the Bayesian approach is getting more and more attention from the industry. At least, Bayesian approach has more information for decision-making, which could potentially help enterprises achieve higher efficiency, shorter period and lower investment. This study also expounds the application of Bayesian statistics in the early R & D on anticancer new drugs, and compares and analyzes its idea and application scenarios with frequentist statistics, aiming to provide macroscopic and systematic reference for all related stakeholders.

## 1 贝叶斯学派与频率学派的比较

贝叶斯学派和频率论学派是统计学的两个重要派系，均可用于对不确定结果进行估计和统计推断，但两者的统计思想存在明显差异^[1, 2]^。频率学派认为世界是确定的，总体参数是一个固定常数，样本信息是随机的; 贝叶斯学派则认为世界是不确定的，总体参数是随机变量，样本信息是固定的。因此，两者在信息整合、统计推断和概率解释方面也不相同。以Ⅱ期单臂试验为例，旨在探索新药A治疗晚期卵巢癌的客观缓解率(objective response rate, ORR)是否高于20%。

频率学派将首先建立假设-新药ORR等于20%，然后完全依托试验数据，以证伪演绎的思路，求出观察到试验数据或更极端数据的可能性(*P*值); 如果单侧*P*值小于2.5%，则拒绝假设，认为新药ORR高于20%。贝叶斯学派将首先基于先验信息建立总体参数的概率分布，然后整合试验数据，求出总体参数的后验分布，判断该参数高于20%的后验概率是否高于预先设定的阈值，如95%显著水平或50%相关水平。

与频率统计相比，贝叶斯统计的概率更易于理解，可直接解释为对研究假设发生的相信程度^[3, 4]^。此外，贝叶斯的主要优势在于允许将专家或既往研究提供的先验信息与试验数据进行整合，每次形成的后验信息又可以作为下一次推断的先验信息，并利用信息进行序贯的自适应决策，因此决策更加高效、更加稳健^[5, 6]^。因为贝叶斯统计需要建立模型，计算及统计过程更复杂; 结果准确性依赖于先验信息的可靠性和可交换性，在确证性试验中的应用受到监管的限制^[7]^。

## 2 肿瘤新药早期研发的新形势与新需求

近些年来，全球肿瘤新药研发依然如火如荼，但依然存在高失败率的风险，尤其是肿瘤早期研发^[8, 9]^。随着研发进展，肿瘤治疗手段不断增加，肿瘤新药研发也逐步从细胞毒药时代过渡为靶向药和免疫药时代，由单药治疗向联合治疗为主转变^[10, 11]^; 以Ⅰ期/Ⅱ期无缝研究、扩展队列研究、平台研究等为代表的更高效、更灵活的适应性设计也备受青睐^[12, 13]^。掌握新形势下肿瘤早期开发的新需求，采用科学有效的设计和方法提升肿瘤新药早期研发成功率，是医药企业共同关注的热点和难点。

### 2.1 肿瘤新药研发从细胞毒药时代过渡为靶向药和免疫药时代

① 与细胞毒药的间歇毒性不同，非细胞毒药通常是每天服用，呈现连续毒性，因此对毒性容忍度可能不同，依据毒性特点及临床需求等特点，对毒性容忍度进行更灵活的设置是肿瘤早期研究的实际需求之一; ②非细胞毒药常见延迟毒性、延迟反应，因此在执行期中监测时，无法通过已观测数据进行期中决策，为避免暂停入组直到所有患者观测到反应所致的试验周期延长，基于贝叶斯方法使用已观测数据提高期中分析决策效率是肿瘤早期试验的实际需求之二; ③非细胞毒药物可能无法达到最大耐受剂量(maximal tolerable dose, MTD)，有效率也不再呈现细胞毒药的单调递增性，既往将毒性和有效性分别考虑、先Ⅰ期再Ⅱ期的传统设计不再适用; 基于风险获益原则，开展Ⅰ期/Ⅱ期无缝设计，构建贝叶斯决策模型以同时权衡有效性-毒性，进而提高早期研发效率是肿瘤早期试验的实际需求之三(图 1)。

**表 1:** 贝叶斯方法和频率论方法的比较 Bayesian versus frequentist paradigms

Item	Frequentist	Bayesian
Statistical parameter	Fixed and unknown	Random variable with probability distributions
Parameter estimation	Totally based on data observed	Combined prior with data observed
Statistical inference	Deductive, calculating the probability of observing a more extreme statistic given the null hypothesis	Inductive, yielding the posterior distribution based on prior and data observed
Probability definition	Probability is defined as a result when a particular design is repeated and infinitum	Probability is defined in relation to one’s pre-existing belief

**图 1:**
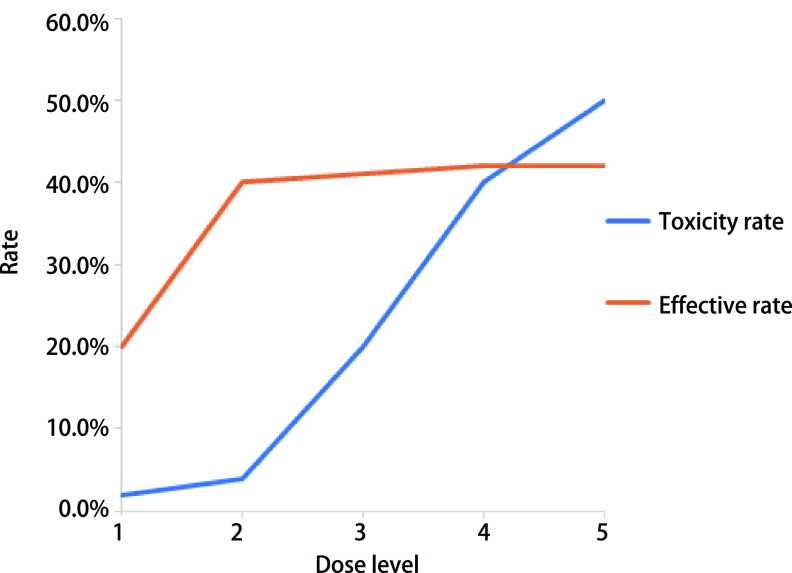
不同剂量水平下肿瘤靶向新药A的毒性和有效性变化 Changes in toxicity and efficacy of anticancer new drug at different dose levels

### 2.2 肿瘤新药研发由单药治疗向联合治疗转变

联合治疗剂量探索面临诸多挑战，剂量探索范围成倍增加。更为重要的是，与单药治疗毒性完全有序相比，联合治疗毒性率和反应率在剂量组合矩阵中通常仅局部有序。举例来说，A_m_B_n_代表药物A剂量水平m和药物B剂量水平n的组合，我们无法知道A_3_B_2_、A_2_B_3_、A_1_B_4_的毒性和有效性的大小关系，组合矩阵中可能存在MTD等高线的情形。基于贝叶斯框架不断更新剂量-毒性曲线后验估计，指导后续患者剂量分配提高早期研发效率是实际需求之四。

### 2.3 传统研究研发模式向现代研发模式的转变

相对而言，传统研发存在高投入、长周期、低效率等不足，采用科学合理的统计设计，实现向现代研发模式转变—低投入、短周期、高效率，得到工业界和监管方等全行业越来越广泛地重视。肿瘤新药早期试验设计中，也越来越常用基于模型或模型辅助的剂量探索设计、Ⅰ期/Ⅱ期无缝设计、多队列扩展、主方案设计等^[13-16]^。借用专家信息、历史数据、早期试验信息，帮助试验决策、减少资源浪费、加速新药研发进程，已成为现代研发模式的共同需求和实践。

## 3 贝叶斯方法在肿瘤新药早期研发的应用与进展

基于上述肿瘤新药早期研发的新趋势和新需求，随着计算机技术的发展，以信息借用、灵活高效为特点的贝叶斯设计方法逐步被学术界提出。随着全球主要监管对药物早期研发的态度开放，贝叶斯方法在肿瘤新药早期研发中的应用也得到工业界的广泛青睐，尤其是在肿瘤新药的剂量探索、疗效探索及主方案设计等领域。

### 3.1 贝叶斯方法助力肿瘤新药剂量探索

基于算法的爬坡设计由于非常简单、易于实施和理解，广泛应用于肿瘤新药剂量探索，以“3+3”为代表; 但是该类设计存在最优剂量准确性不佳、低剂量暴露人数多、统计性能低等诸多弊端，无法满足肿瘤新药早期研发新形势下的新需求。为提高正确选择MTD的概率，借用信息、建立剂量毒性曲线关系模型，进而更好地帮助剂量爬坡及决策可优效提高研发效率，保护受试者安全。近些年来，以mTPI、BOIN设计为代表贝叶斯模型辅助设计和以持续再评估法为代表的贝叶斯模型设计逐步得到应用^[17-19]^。模型辅助决策因为集合了模型设计优良的统计性能和基于算法设计简单易行的优点，应用越来越广泛。针对联合用药试验、延迟毒性、同时考虑药物毒性和有效性等新需求，基于贝叶斯方法的BOIN拓展设计及其工具近些年也得到了工业界的追捧^[19, 20]^。

### 3.2 贝叶斯方法助力肿瘤新药疗效探索

建立新药在目标人群总体疗效参数的先验分布; 实时监测试验数据，根据试验观察到的数据，计算药物达到与目标疗效的后验概率; 基于预设标准，及时终止无效队列，结束优效队列进入下一阶段，可有效减少资源浪费、加速新药研发进程。由于操作简单，基于频率派的Simon两阶段已被广泛应用于Ⅱ期肿瘤药物临床试验中，但该设计也存在一些局限性^[21]^。通常，Simon两阶段仅允许一次期中分析、仅适用于二分类结局研究、无法借用历史数据和先验信息。更加灵活和高效的基于贝叶斯的Ⅱ期试验设计方法也被提出，可以实现组间信息借用，更稳健地进行早期疗效的实时监测、预测和决策，包括MUCE队列拓展、贝叶斯最优Ⅱ期设计、针对有延迟反应多重填补贝叶斯设计等^[14, 22, 23]^。

### 3.3 贝叶斯方法助力主方案设计

平台设计是一类能够同时评估多种候选药物、多种肿瘤或分子亚型疗效的设计，包括篮子试验、伞式试验和平台试验^[24, 25]^，研究集中管理、统一决策。基于贝叶斯方法科学借用各试验组间信息，可有效节省样本量，学术界先后提出了贝叶斯分层模型(Bayesian Hierarchical Model, BHM)，实现在篮子间自适应地借用信息、允许多次期中分析^[26, 27]^; MIDAS(multi-candidate iterative design with adaptive selection)贝叶斯Ⅱ期试验设计，允许共用对照组，借用组间信息，以高效、无缝地方式持续筛选大量候选药物; 贝叶斯药物组合平台设计，实现自适应跨组合的信息借用以高效识别有效组合。

## 4 总结与讨论

近年来，我国临床试验的实施和监管已实现与国际规范的逐步接轨，对前沿理念和设计方法在新药、新技术早期研发中应用的态度也更加的开放，尤其是在医疗器械产品开发中^[28]^。在肿瘤新药研发高风险、高收益的双重背景下，面向肿瘤新药早期研发新形势下的难点问题和新的需求，贝叶斯临床试验设计因其高效性和灵活性得到工业界和学术界的广泛重视，尤其是在国外已经有较多的实践应用。

值得注意的是，基于贝叶斯方法的后验概率均值本质上是先验期望和观测数据期望的加权平均。随着观测数据样本量不断增加，基于贝叶斯方法所得概率会和频率派方法趋于一致。也就是说，在大样本研究中，贝叶斯方法借用信息使得结果更加稳健的优势将消失; 由于贝叶斯方法的计算和建模更加复杂，因此，不推荐在确证性试验等大样本研究中使用贝叶斯方法。再者，在确证性研究中，监管更加重视多重性问题和I类错误控制，尚未有监管在新药确证性试验中接受企业采用贝叶斯方法。早期小样本研究是目前贝叶斯方法的主要适宜场景。

贝叶斯方法通过借用先验信息使得早期试验及其决策更加高效。先验信息的可交换性至关重要，即历史数据、专家判定的信息适用于本研究决策参考的程度。举例来说，篮子试验是一类在具有相同突变、不同组织类型的患者中评估一种药物的设计，多为Ⅱ期早期疗效探索研究。该类设计的实际挑战是尽管患者具有相同突变，但疗效反应有所不同。完全借用组间信息无疑会提高统计检验效能，但也会导致I类错误的膨胀; 完全不借用信息，能很好的控制I类错误，但统计效能会降低。采用BHM等方法，基于数据观测到的组间异质性，自适应地确定借用信息的程度对准确估计疗效至关重要。

研究存在一些不足。限于篇幅，研究对肿瘤早期研发的新趋势与新需求、贝叶斯方法应用概况不够详尽，比如贝叶斯方法在生物标志物富集设计、个性化剂量探索方面的应用。总体来说，本研究以肿瘤新药早期研发的新趋势、新需求为导向，科学阐述了贝叶斯方法的进展及应用价值，并与频率派的理念、应用场景进行了比较分析，可为医药研发的所有从业人员提供宏观、系统的参考。
